# GLP‐1 suppresses glucagon secretion in human pancreatic alpha‐cells by inhibition of P/Q‐type Ca^2+^ channels

**DOI:** 10.14814/phy2.13852

**Published:** 2018-09-05

**Authors:** Reshma Ramracheya, Caroline Chapman, Margarita Chibalina, Haiqiang Dou, Caroline Miranda, Alejandro González, Yusuke Moritoh, Makoto Shigeto, Quan Zhang, Matthias Braun, Anne Clark, Paul R. Johnson, Patrik Rorsman, Linford J. B. Briant

**Affiliations:** ^1^ Oxford Centre for Diabetes, Endocrinology and Metabolism, Radcliffe Department of Medicine University of Oxford Oxford United Kingdom; ^2^ Institute of Neuroscience and Physiology Metabolic Research Unit University of Göteborg Göteborg Sweden; ^3^ Nuffield Department of Surgery University of Oxford John Radcliffe Hospital Oxford United Kingdom; ^4^ NIHR Oxford Biomedical Research Centre Oxford United Kingdom; ^5^ Department of Computer Science University of Oxford Oxford United Kingdom

**Keywords:** cAMP, cyclic adenosine monophosphate, GLP‐1, glucagon‐like peptide 1, K_ATP_, potassium ATP channel, SST, somatostatin, T2DM, Type 2 diabetes mellitus

## Abstract

Glucagon is the body's main hyperglycemic hormone, and its secretion is dysregulated in type 2 diabetes mellitus (T2DM). The incretin hormone glucagon‐like peptide‐1 (GLP‐1) is released from the gut and is used in T2DM therapy. Uniquely, it both stimulates insulin and inhibits glucagon secretion and thereby lowers plasma glucose levels. In this study, we have investigated the action of GLP‐1 on glucagon release from human pancreatic islets. Immunocytochemistry revealed that only <0.5% of the *α*‐cells possess detectable GLP‐1R immunoreactivity. Despite this, GLP‐1 inhibited glucagon secretion by 50–70%. This was due to a direct effect on *α*‐cells, rather than paracrine signaling, because the inhibition was not reversed by the insulin receptor antagonist S961 or the somatostatin receptor‐2 antagonist CYN154806. The inhibitory effect of GLP‐1 on glucagon secretion was prevented by the PKA‐inhibitor Rp‐cAMPS and mimicked by the adenylate cyclase activator forskolin. Electrophysiological measurements revealed that GLP‐1 decreased action potential height and depolarized interspike membrane potential. Mathematical modeling suggests both effects could result from inhibition of P/Q‐type Ca^2+^ channels. In agreement with this, GLP‐1 and *ω*‐agatoxin (a blocker of P/Q‐type channels) inhibited glucagon secretion in islets depolarized by 70 mmol/L [K^+^]_o_, and these effects were not additive. Intracellular application of cAMP inhibited depolarization‐evoked exocytosis in individual *α*‐cells by a PKA‐dependent (Rp‐cAMPS‐sensitive) mechanism. We propose that inhibition of glucagon secretion by GLP‐1 involves activation of the few GLP‐1 receptors present in the *α*‐cell membrane. The resulting small elevation of cAMP leads to PKA‐dependent inhibition of P/Q‐type Ca^2+^ channels and suppression of glucagon exocytosis.

## Introduction

Glucagon is the most important hyperglycemic hormone of the body (Lefebvre 1996; Cryer 2002). In both type 1 and type 2 diabetes mellitus, hyperglycemia results from a combination of insufficient insulin secretion and oversecretion of glucagon (Dunning et al. 2005).

Glucagon is secreted from the *α*‐cells of the pancreatic islets. Both intrinsic (Vieira et al. 2007; Zhang et al. 2013; Li et al. 2015) and paracrine (Gromada et al. 2007; Da Silva Xavier 2018) mechanisms have been proposed in the regulation of glucagon secretion by glucose. Among the intrinsic mechanisms, it has been postulated that glucose inhibits glucagon secretion via closure of plasmalemmal K_ATP_‐channels and membrane potential‐dependent inactivation of the ion channels involved in *α*‐cell electrical activity and exocytosis (Gopel et al. 2000; MacDonald et al. 2007; Zhang et al. 2013). Paracrine regulation may involve the release of factors by neighboring *β*‐ and *δ*‐cells; possible paracrine factors include GABA (Rorsman et al. 1989), insulin (Banarer et al. 2002; Ravier and Rutter 2005), Zn^2+^ (Ishihara et al. 2003), and somatostatin (Pipeleers et al. 1985).

The incretin hormone glucagon‐like peptide 1 (GLP‐1) is secreted from gastrointestinal L‐cells in response to nutrients (Holst 2007; Gribble and Reimann 2016). This hormone has attracted much attention because of its capacity to enhance insulin release and reduce plasma glucose levels (Drucker 2006; Holst 2007). Several GLP‐1 receptor agonists have been developed for the treatment of type 2 diabetes mellitus (T2DM; see review by Andersen et al. (2018)). As well as reducing postprandial (Kolterman et al. 2003) and fasting (Buse et al. 2009) plasma glucose, these agonists have been observed to reduce body weight (Raccah et al. 2014) and improve cardiovascular outcomes (Nissen and Wolski 2007) in T2DM.

Despite the numerous advantageous effects of GLP‐1 agonists, the precise mechanism by which GLP‐1 reduces plasma glucose remains obscure. In addition to reducing hepatic glucose production, gastrointestinal motility and satiety (see reviews by Barrera et al. (2011) and Campbell and Drucker (2013)), GLP‐1 influences hormone secretion from pancreatic islets (Holst 2007). The beneficial effect of GLP‐1 on islet hormone secretion is twofold; GLP‐1 has the capacity to both enhance insulin and reduce glucagon release (Nauck et al. 1993). Importantly, the reciprocal modulation of insulin and glucagon secretion contributes equally to the ability of GLP‐1 to lower plasma glucose (Hare et al. 2010). In type 1 diabetic patients with minimal *β*‐cell function, GLP‐1 is still able to lower fasting plasma glucose concentrations, presumably via reducing plasma glucagon concentrations (Creutzfeldt et al. 1996). The action of GLP‐1 on *β*‐cells has been studied in mouse, revealing the mechanism by which insulin secretion is stimulated (Holz et al. 1995; Dyachok et al. 2006) and clearly demonstrating that *β*‐cell function is an important component of the response to exogenous GLP‐1 (Smith et al. 2014).

In contrast, the mechanism(s) by which GLP‐1 inhibits glucagon secretion remains obscure, with studies in rodent islets indicating that GLP‐1 could act via an intrinsic (De Marinis et al. 2010) or a paracrine (de Heer et al. 2008) mechanism. We have previously reported that GLP‐1 inhibits glucagon secretion in mouse by inhibition of the high voltage‐activated Ca^2+^ channels linked to glucagon exocytosis (De Marinis et al. 2010). Whether these observations can be extended to human *α*‐cells remains unknown but such information is needed for full understanding of GLP‐1's clinical effects. Here, we have examined the effects of GLP‐1 on glucagon secretion and *α*‐cell function in isolated human pancreatic islets by a combination of immunocytochemistry, hormone secretion measurements, Ca^2+^ imaging and electrophysiological recordings. Our data suggest that GLP‐1 directly suppresses *α*‐cells by inhibiting P/Q‐type voltage‐gated Ca^2+^ channels.

## Materials and Methods

### Ethical approval

All experiments were conducted in accordance with the University of Oxford ethical guidelines, and were approved by the local Ethics Committees. Human pancreatic islets were isolated (with ethical approval and clinical consent) at the Diabetes Research and Wellness Foundation Human Islet Isolation Facility (OCDEM, Oxford, UK) from the pancreases of 21 nondiabetic donors. Donors (10 females, 11 males) were on average 48 years old (range 19–61) with a BMI of 28 (range 21–37).

For species comparison of gene expression, one set of experiments were done in mouse islets. This work was conducted in accordance with the UK Animals Scientific Procedures Act (1986) and University of Oxford and Gothenburg University ethical guidelines, and were approved by the respective local Ethics Committees.

### Isolation of mouse islets

For analysis of expression of cAMP effectors, NMRI mice (12 weeks old) were killed by cervical dislocation (Schedule 1 procedure). Pancreatic islets were isolated by liberase digestion and manually picked. Islets were used acutely and were, pending the experiments, maintained in tissue culture for <24 h in RPMI medium containing 10 mmol/L glucose prior to the measurements.

### Reagents

GLP‐1 (7–36) amide was from Bachem (Weil am Rhein, Germany). We used GLP‐1 at a concentration of 10 nM, in keeping with other islet cell studies (Bode et al. 1999; Tsuboi et al. 2003; de Heer et al. 2008; De Marinis et al. 2010; Shigeto et al. 2015; Traub et al. 2017). *ω*‐Agatoxin was purchased from the Peptide Institute (Minoh‐shi Osaka, Japan), 8‐Br‐Rp‐cAMPS (Rp‐cAMPS) from BioLog Life Science Institute (Bremen, Germany) and CYN154806 from Tocris Bioscience (Bristol, UK). Adrenaline, diazoxide, exendin (9–39), S961, and forskolin were all from Sigma‐Aldrich Company Ltd. (Gillingham, UK). When test substances were dissolved in DMSO, an equal concentration (<0.1% v/v) of the solvent was present under all control conditions.

### Measurements of islet hormone secretion

Prior to secretion experiments, human islets were maintained in culture for up to 48 h in RPMI containing 5 mmol/L glucose. Experiments were conducted using batches of 13–15 hand‐picked and size‐matched islets per tube (in triplicate). We note that glucagon secretion exhibits variability between preparations, probably due to differences in the quality, function, and donor details of human islets (Ihm et al. 2006; Hanson et al. 2010; Kayton et al. 2015). To circumvent these confounds, each donor was used as its own control when testing the effect of a compound.

Islets were washed twice in RPMI prior to preincubation in Krebs‐Ringer buffer (KRB) containing 2 mg/mL BSA (S6003, Sigma‐Aldrich) and 3 mmol/L glucose for 1 h at 37°C. Following this, islets were incubated in 0.3 mL KRB with 2 mg/mL BSA, supplemented with various glucose concentrations or compounds (e.g., 10 nmol/L GLP‐1), depending on the experimental condition. After each incubation, the supernatant was removed and quickly frozen and stored at −80°C. Insulin, glucagon, and somatostatin (SST) were measured by radioimmunoassay (EURIA, Euro Diagnostic, Malmo, Sweden).

### Measurements of islet cAMP

Human islets were preincubated for 2 h at 37°C in RPMI containing no glucose and supplemented with 0.05% BSA. The islets were then incubated at 37°C for 30 min in 1 mL of KRB‐BSA medium containing 1 mmol/L glucose and 100 μmol/L isobutyl methylxanthine (IBMX), supplemented with the various forskolin concentrations. In membrane preparations, forskolin activates cAMP with an IC_50_ of ~1 *μ*mol/L (Seamon et al. 1981). To investigate the role of cAMP in intact islets, we used concentrations of forskolin ranging from 1 nmol/L to 1 *μ*mol/L. An aliquot of the supernatant was removed immediately after incubation and frozen for glucagon assay. cAMP was extracted by adding 400 μL of ice‐cold sodium acetate buffer (50 mmol/L, pH 6.2) to the islets. Samples were boiled for 10 min before being stored at −80°C. cAMP levels were determined by RIA (Amersham Pharmacia Biotech, Braunschweig, Germany).

### Measurements of *α*‐cell membrane currents, membrane capacitance, and electrical activity

All electrophysiological recordings were performed using an EPC‐9 patch‐clamp amplifier (HEKA Electronics, Lambrecht/Pfalz, Germany) and Pulse software (version 8.50), as previously described (Briant et al. 2018a). For capacitance measurements, human islets were dissociated into single cells and plated on plastic 35 mm tissue culture dishes (Sarstedt Inc.) and maintained in an incubator in 7.5 mmol/L glucose. The extracellular medium contained (in mmol/L) 118 NaCl, 20 tetraethylammonium chloride (TEA‐Cl), 5.6 KCl, 2.6 CaCl_2_, 1.2 MgCl_2_, 5 HEPES (pH 7.4 with NaOH), 6 mmol/L glucose, and 0.1 *μ*g/mL TTX. The pipette solution contained (in mmol/L) 125 Cs‐glutamate, 10 CsCl, 10 NaCl, 1 MgCl_2_, 3 Mg‐ATP, 5 HEPES (pH 7.15 with CsOH), and 0–0.1 mmol/L cAMP. Cell identity was confirmed by immunocytochemistry (Braun et al. 2009). Exocytosis was measured as increases in cell capacitance in response to 500 msec depolarizations from −70 to 0 mV using the Sine‐DC technique.

Membrane potential was recorded from *α*‐cells in intact islets using the perforated patch whole‐cell technique (Gopel et al. 2000). During these experiments, the islets were perfused with an extracellular medium composed of (mmol/L) 140 NaCl, 3.6 KCl, 1.3 CaCl_2_, 0.5 MgSO_4_, 0.5 Na_2_H_2_PO_4_, 5 NaHCO_3_, and 10 HEPES (pH 7.40 with NaOH), The pipette solution contained (in mmol/L) 76 K_2_SO_4_, 10 NaCl, 10 KCl, 1 MgCl_2_, and 5 HEPES (pH 7.35 with CsOH). Perforation was achieved by addition of amphotericin B at a final concentration of 60 *μ*g/mL. Interspike and peak action potential voltages were measured by the template search algorithm of ClampFit (version 9.2.0.11, Axon Instruments Inc.). The cells were labeled by injection of biocytin (1 mg/mL included in electrode solution) at the end of the experiment and cell identity was subsequently confirmed by immunocytochemistry (Zhang et al. 2007).

### Quantitative imaging of Ca^2+^

Time‐lapse imaging of the intracellular Ca^2+^ concentration ([Ca^2+^]_i_) in islets was performed on the inverted Zeiss AxioVert 200 microscope, equipped with the Zeiss LSM 510‐META laser confocal scanning system, using a 403/1.3 NA objective. Human islets were loaded with 6 mmol/L of the Ca^2+^‐sensitive dye Fluo‐4 for 90 min before being transferred to a recording chamber. Islets were then continuously perfused with bath solution (same solution as described for patch‐clamp electrophysiology, above) at a rate of 200 *μ*L/min. Fluo‐4 was excited at 488 nm and fluorescence emission imaged at 530 nm. The pinhole diameter was kept constant, and frames of 256 × 256 pixels were taken every 1–3 sec. *α*‐cells were identified by the presence of oscillations in [Ca^2+^]_i_ in low (3 mmol/L) glucose and an excitatory response to adrenaline (Gromada et al. 1997; Hamilton et al. 2018).

### Mathematical modeling of *α*‐cell membrane potential

All simulations were conducted in the simulation environment NEURON using CVODE and a 25 *μ*sec timestep (Carnevale and Hines 2006). The equation describing membrane potential $\left(V_{M}\right)$ in the human *α*‐cell model was:

$$\begin{array}{cc} C_{\mathrm{cell}}\frac{dV_{M}}{dt}= & -\left(\right.I_{\mathrm{CaL}}+I_{\mathrm{CaN}}+I_{\mathrm{CaT}}+I_{P/Q}+I_{\mathrm{Na}}+I_{K}\\ & +I_{\mathrm{KATP}}+I_{\mathrm{KA}}+I_{L}) \end{array}$$

where: *C*_cell_ is the cell capacitance; *I*_CaL_, *I*_CaN_, *I*_CaT_ and *I*_CaP‐Q_ are L‐, N‐, T‐type, and P/Q‐type voltage‐dependent Ca^2+^ currents, respectively; *I*_Na_ is a voltage‐dependent Na^+^ current; *I*_K_ is a delayed rectifier K^+^ current; *I*_KA_ is an A‐type voltage‐dependent K^+^ current; *I*_K(ATP)_ is an ATP‐sensitive K^+^ current; *I*_L_ is a leak current. This model is adapted from a model of a mouse *α*‐cell recently described (Briant et al. 2016, 2017). The adaptations were to accommodate the known differences in ion channel properties between mouse and human *α*‐cells: in particular, an increase in P/Q‐type Ca^2+^ current and a decrease in Na^+^ current (Ramracheya et al. 2010). The model is made freely available on request.

### Immunocytochemistry

Human islets were fixed in 10% neutral‐buffered formalin, dehydrated, and processed for paraffin wax embedding and sectioning (3 *μ*m). GLP‐1R immunoreactivity was detected using rabbit anti‐GLP‐1R (LS‐A‐1205 and LS‐A‐1206, Lifespan, 1:100 and 1:750, respectively) antibodies. Cell identity (*α*,* β,* or *δ*) was established by costaining for hormones. Islets were permeabilized with 0.3% Triton X‐100 and then incubated at 4°C with primary antibodies for 4–12 h (guinea pig anti‐insulin (Abcam, Cambridge, UK), sheep anti‐glucagon (Sigma‐Aldrich, St Louis, MO) and rabbit anti‐somatostatin (Vector Labs, Burlingame, CA). After washing with PBS, the islets were incubated for 1 h in secondary antibodies (Alexa 633 goat anti‐guinea pig (insulin), Alexa 405 goat anti‐mouse (glucagon) and Alexa 543 goat anti‐rabbit (somatostatin)). Islets were imaged on a confocal microscope (Axioskop 2 upright microscope fitted with a Zeiss LSM 510 meta confocal and a chameleon multiphoton module). Quantifying the colocalisation of GLP‐1R with insulin, glucagon and, SST was conducted manually by counting the number of coexpressing cells.

### RNA extraction and quantitative RT‐PCR

Gene expression was analyzed by quantitative RT‐PCR in islets from three 12 week‐old NMRI mice and four human donors. Total RNA was isolated using a combination of TRIzol and PureLink RNA Mini Kit (Ambion, Thermofisher Scientific). On column DNase treatment was included to eliminate DNA contamination. cDNA was synthesized from 500 ng of total RNA using the High Capacity RNA‐to‐cDNA kit (Applied Biosystems, Thermofisher Scientific). Real time qPCR was performed using SYBR Green detection and gene specific QuantiTect Primer Assays (Qiagen) on a 7900HT Applied Biosystems analyser. All reactions were run in triplicates. Relative expression was calculated using ΔCt method, with GAPDH and PPIA used as reference genes.

### Data analysis

All data are reported as mean ± SEM, unless otherwise stated. Statistical significance was defined as *P* < 0.05. All statistical tests were conducted in Prism 5 (GraphPad Software, San Diego, CA). For two groupings, a *t* test was conducted. If the data were nonparametric, a Mann–Whitney test was conducted. For more than two groupings, a one‐way ANOVA was conducted. If there were two independent variables, a two‐way ANOVA was conducted. If the data passed normality criteria (D'Agostino's test of normality and Bartlett's test of equal variances), a parametric test was conducted with the appropriate post hoc test (Tukey or Student Neumann Keuls). If the normality criteria were not met, a Kruskal–Wallis test with Dunn's multiple comparison test was conducted. For secretion data, a minimum of two human donors were used and each replicate was considered an individual experiment.

## Results

### GLP‐1 receptors are weakly expressed in *α*‐cells

**Figure 1:**
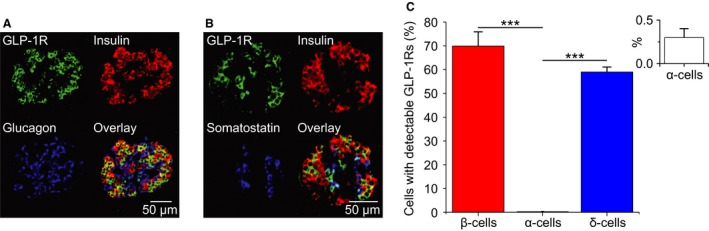
Human *α*‐cells exhibit low expression levels of GLP‐1R. (A, B) Immunostaining of human islets for the GLP‐1R, insulin, glucagon, and SST. (C) % of insulin‐, glucagon‐, and SST‐positive cells that express GLP‐1R. Inset in (C): zoom for glucagon positive cells. *β*‐, *α*‐, and *δ*‐cell data were collected from 162 and 173 islets, respectively, from three human donors. One‐way ANOVA;* P* < 0.001 = ***.

Immunocytochemistry was used to investigate GLP‐1R expression levels in cells within intact human pancreatic islets (Fig. 1). GLP‐1R immunoreactivity was detectable in 70% of the insulin‐positive *β*‐cells (Fig. 1A). Only 0.3% of the glucagon‐positive *α*‐cells were positive for GLP‐1R (Fig. 1C). The somatostatin‐positive *δ*‐cells exhibited almost as strong GLP‐1R immunoreactivity as the *β*‐cells (60%; Fig. 1B). The low expression of GLP‐1R in human *α*‐cells is in general agreement with previous findings for protein (Tornehave et al. 2008), bulk RNA‐seq (Blodgett et al. 2015), and single‐cell RNA‐seq (Segerstolpe et al. 2016) data of GLP‐1R expression in human islets.

### GLP‐1 exerts a strong inhibitory effect on glucagon secretion from human islets

**Figure 2:**
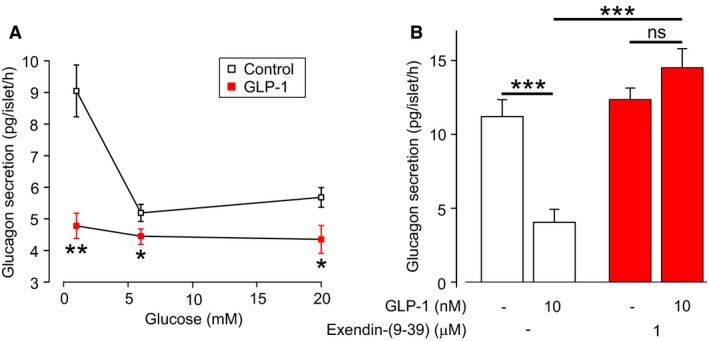
GLP‐1 inhibits glucagon secretion from human islets. (A) Glucagon secretion from human islets in the presence (filled squares) or absence (open square) of GLP‐1 (10 nmol/L), at various glucose concentrations. Experiments in triplicate from eight human donors. Two‐way ANOVA,* P* < 0.05 = *, *P* < 0.01 = **. (B) Effect of the GLP‐1R antagonist exendin (9–39) on glucagon secretion, in the presence or absence of GLP‐1. Experiments in triplicate from two human donors. One‐way ANOVA,* P* < 0.001 = ***, ns=not significant. Glucose concentration is 1 mmol/L.

We examined the effects of GLP‐1 on glucagon secretion from human islets (Fig. 2). The ability of GLP‐1 to inhibit glucagon secretion did not depend on the glucose concentration, as GLP‐1 (10 nmol/L) could inhibit glucagon secretion in 1, 6, and 20 mmol/L glucose (Fig. 2A). However, the strongest inhibition occurred in 1 mmol/L glucose (Fig. 2A). The inhibitory effect of GLP‐1 on glucagon secretion was prevented by the GLP‐1R antagonist exendin‐(9–39) (Fig. 2B).

### The inhibitory effect of GLP‐1 on glucagon secretion is not mediated by insulin or somatostatin

**Figure 3:**
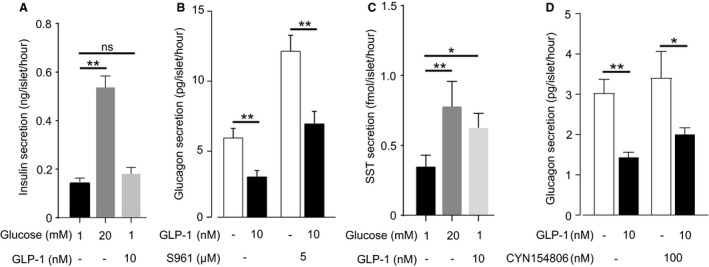
The inhibition of glucagon secretion by GLP‐1 is not due to paracrine mechanisms. (A) Insulin secretion in the presence of high glucose (20 mmol/L), and low glucose (1 mmol/L) with and without GLP‐1. Experiments in triplicate from seven human donors. (B) Effect of the insulin receptor antagonist S961 on GLP‐1 mediated inhibition of glucagon secretion. Experiments in triplicate from seven human donors. Glucose concentration is 1 mmol/L. (C) Same as (A) but for SST secretion. Experiments in triplicate from seven human donors. (D) Same as (B) but for the SST receptor type 2 antagonist, CYN154806. Experiments in triplicate from four human donors. One‐way ANOVA,* P* < 0.05 = *, *P* < 0.01 = **, ns = not significant.

It has been suggested that because of the low expression of GLP‐1Rs in *α*‐cells, the effects of GLP‐1 on glucagon release are instead mediated by insulin or SST released from neighboring *β*‐ and *δ*‐cells, respectively (Orskov et al. 1988; Fehmann et al. 1995; Herrmann et al. 1995; de Heer et al. 2008). Although our own experiments in mouse islets led to the conclusion that the effects of GLP‐1 on glucagon secretion are mediated by direct effects on *α*‐cells (De Marinis et al. 2010), other studies indicate a role for paracrine mediators (de Heer et al. 2008). We therefore tested whether the inhibitory action of GLP‐1 on glucagon secretion in human islets may be mediated by a paracrine mechanism (Fig. 3). When GLP‐1 was applied at 1 mmol/L glucose, insulin secretion was not changed (*P* = 0.36; Fig. 3A), suggesting that the suppression of glucagon secretion by GLP‐1 is not due to changes in insulin signaling. In support of this, in the presence of S961, a high‐affinity peptide insulin receptor antagonist (Schaffer et al. 2008), basal glucagon secretion was increased by 100%, but GLP‐1 retained an inhibitory effect on glucagon secretion (Fig. 3B). SST is released from islet *δ*‐cells and has a powerful inhibitory influence on glucagon secretion (Pipeleers et al. 1985; Klaff and Taborsky 1987). We observed that SST secretion was increased by 150% in response to GLP‐1 (*P* = 0.04; Fig. 3C). The ability of GLP‐1 to stimulate SST secretion at 1 mmol/L glucose might be a consequence of human *δ*‐cells being electrically active at this glucose concentration (Braun et al. 2009) combined with their high GLP‐1R density (Fig. 1). In human *α*‐cells, the dominant SST receptor is SSTR2 (Kailey et al. 2012). We therefore explored whether the inhibitory effect of GLP‐1 on glucagon secretion is mediated by SST using the SSTR2 antagonist CYN154806 (Fig. 3D). In the presence of the SSTR2 antagonist, basal glucagon secretion was marginally higher than under control conditions but GLP‐1 remained inhibitory. There was a tendency to a reduction of this inhibitory effect in the presence of CYN154806, but this effect was not significant (*P* = 0.7431). Together, these data suggest that GLP‐1 inhibits glucagon secretion via a direct action on *α*‐cells, despite the low expression of GLP1Rs in *α*‐cells.

### GLP‐1 inhibits glucagon secretion by a PKA‐dependent mechanism

**Figure 4:**
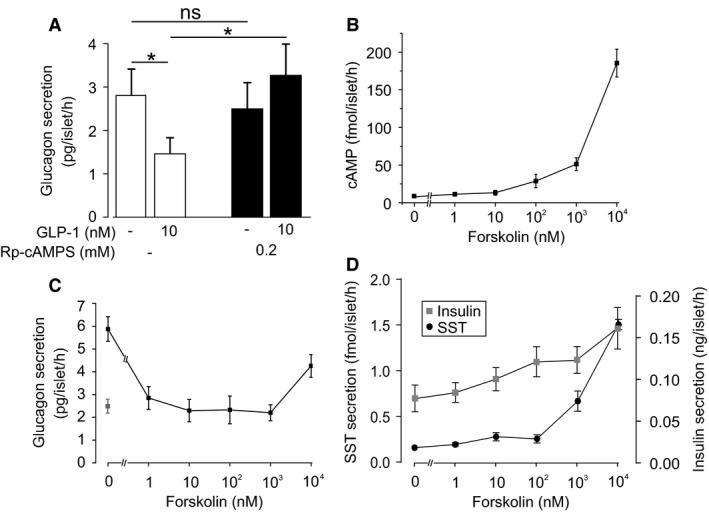
GLP‐1 inhibits glucagon secretion by a PKA‐dependent mechanism. (A) Glucagon secretion from human islets in response to GLP‐1, preincubated with the PKA‐inhibitor 8‐Br‐Rp‐cAMPS (or a DMSO control). Experiments in triplicate from four human donors. One‐way ANOVA;* P* < 0.05 = *. Glucose concentration is 1 mmol/L. (B) Whole‐islet cAMP from human islets incubated in various concentrations of the adenylate cyclase stimulator forskolin. Experiments in triplicate from five human donors. Glucose concentration is 1 mmol/L. (C) Same as (A) but measuring islet glucagon secretion. Gray data‐point represents glucagon secretion in the presence of 10 nmol/L GLP‐1 (without forskolin). Experiments in triplicate from two human donors. Glucose concentration is 1 mmol/L. (D) Same as (C) but SST (•) and insulin (▪) secretion. Experiments in triplicate from three human donors. Glucose concentration is 1 mmol/L.

Binding of GLP‐1 to GLP‐1R activates adenylyl cyclase, catalyzing the conversion of ATP into cyclic adenosine monophosphate (cAMP; Doyle and Egan (2007)). In mouse *α*‐cells, cAMP has multiple effects that contribute to glucagon secretion (Tengholm and Gylfe 2017) and GLP‐1 has been reported to increase cytoplasmic cAMP in a subset of *α*‐cells (Tian et al. 2011). We therefore investigated whether the glucagon lowering effect of GLP‐1 is cAMP‐ and PKA‐dependent (Fig. 4).

Pharmacological blockade of PKA by pretreatment of islets with the membrane‐permeable PKA‐inhibitor 8‐Br‐Rp‐cAMPS (0.2 mmol/L) prevented the inhibitory effect of GLP‐1 on glucagon secretion (Fig. 4A). Notably, there was no effect of PKA inhibition on basal glucagon secretion at 1 mmol/L glucose.

We compared the effect of GLP‐1 on glucagon secretion with those of the adenylate cyclase activator forskolin. Forskolin at concentrations of 1 nmol/L ‐ 1 *μ*mol/L increased islet cAMP content (Fig. 4B) and inhibited glucagon secretion by >50% (Fig. 4C). In mouse islets, high concentrations of forskolin stimulate glucagon secretion (De Marinis et al. 2010). However, in human islets even a concentration as high as 10 *μ*mol/L failed to produce a net stimulation of glucagon secretion (although the inhibitory effect was less pronounced than at lower concentrations). We note that although Edlund et al. (2017) demonstrated that 10 *μ*mol/L forskolin stimulates glucagon, these secretion studies were conducted at 2.8 mmol/L glucose (rather than at 1 mmol/L, as in our experiments). Finally, inhibition of glucagon secretion did not correlate with any major stimulatory effects on insulin and somatostatin secretion (Fig. 4D).

### Effect of GLP‐1 on [Ca^2+^]_i_ and electrical activity in *α*‐cells

**Figure 5:**
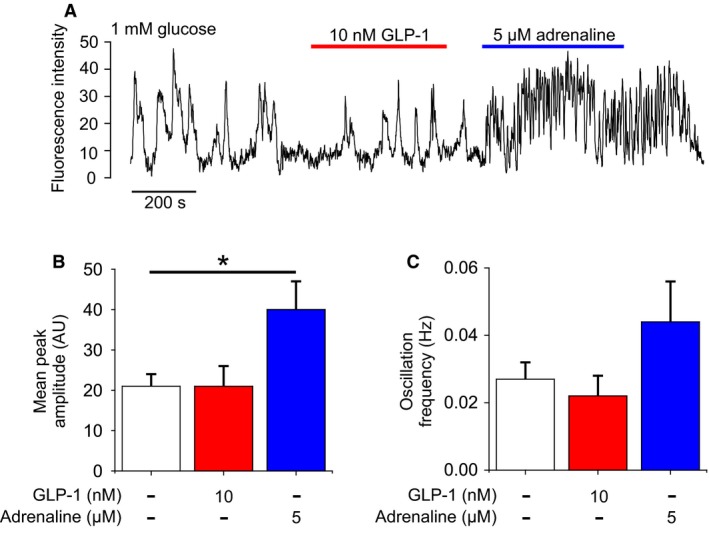
GLP‐1 does not modulate intracellular Ca^2+^ dynamics. (A) Time‐series of intracellular Ca^2+^ from a human *α*‐cell (identified by adrenaline response; Hamilton et al. (2018)). Responses to GLP‐1 (10 nmol/L) and adrenaline (5 *μ*mol/L) in the presence of 1 mmol/L glucose. (B) Amplitude of Ca^2+^ transients in 1 mmol/L glucose (1G), with 10 nmol/L GLP‐1 or 5 *μ*mol/L adrenaline. 32 *α*‐cells, 5 islets from three human donors. (C) Frequency of Ca^2+^ transients in 1 mmol/L glucose (1G), with 10 nmol/L GLP‐1 or 5 *μ*mol/L adrenaline (adr). 32 *α*‐cells, five islets from three human donors.

Glucagon exocytosis is a Ca^2+^‐dependent process (Gromada et al. 1997). We explored whether GLP‐1 mediates its inhibitory effect by reduction of cytoplasmic Ca^2+^ ([Ca^2+^]_i_). Human *α*‐cells can be identified by the occurrence of spontaneous [Ca^2+^]_i_ oscillations in 1 mmol/L glucose that are stimulated by adrenaline (Hamilton et al. 2018). Whereas GLP‐1 was without any major effect on [Ca^2+^]_i_ oscillation amplitude (Fig. 5A and B) or frequency (Fig. 5A and C), a strong stimulation was produced by adrenaline (Fig. 5A and B).

**Figure 6:**
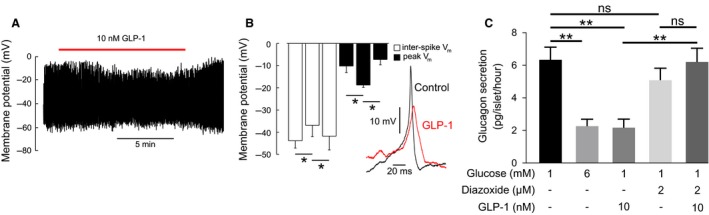
GLP‐1 reduces *α*‐cell action potential amplitude. (A) Membrane potential (*V*_m_) recording from human *α*‐cell in 1 mmol/L glucose. Application of 10 nmol/L GLP‐1 as indicated. (B) Action potential morphology in the presence or absence (control) of GLP‐1 (10 nmol/L). For each condition, an average action potential waveform was computed, and this was used to compute the inter‐spike membrane potential and peak potential of the spike. Repeated measures one‐way ANOVA,* P* < 0.05 = *. 3–4 *α*‐cells from two human donors. (C) Glucagon secretion in response to GLP‐1 in the presence or absence of diazoxide. One‐way ANOVA,* P* < 0.01 = **. Experiments in triplicates from three human donors.

*α*‐cells are electrically active and utilize these electrical signals to drive glucagon exocytosis (Barg et al. 2000), with the exocytotic capacity strongly dependent on action potential amplitude (Zhang et al. 2013). We therefore investigated the effects of GLP‐1 on electrical activity recorded from *α*‐cells in intact human pancreatic islets (Fig. 6A). GLP‐1 did not affect action potential frequency (data not shown), but instead had a consistent lowering effect on the amplitude of the action potentials, via a reduction of action potential peak and interspike voltage (Fig. 6B).

How does GLP‐1 inhibit action potential height? Given that GLP‐1 inhibits K_ATP_ channels in *β*‐cells (Holz et al. 1993; Gromada et al. 1998; Light et al. 2002), we explored whether the effects on *α*‐cell electrical activity may also be mediated by K_ATP_ channel closure. In apparent agreement with this hypothesis, the K_ATP_ channel activator diazoxide (2 *μ*mol/L) fully prevented the inhibitory effect of GLP‐1 on glucagon secretion (Fig. 6C).

### GLP‐1 inhibits glucagon secretion independent of membrane potential

**Figure 7:**
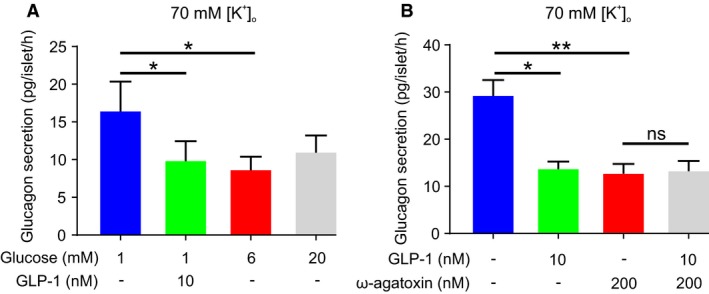
GLP‐1 inhibits glucagon secretion independent of membrane potential. (A) Membrane potential‐independent effects of GLP‐1 and glucose on glucagon secretion. Glucagon secretion was measured in the presence of high extracellular K^+^ (70 mmol/L), which depolarizes *α*‐cell membrane potential and therefore affords the study of membrane potential‐independent effects. Experiments in triplicate from three human donors. One‐way RM ANOVA;* P* < 0.05 = *. (B) Same as in (A) but in the presence and absence of the P/Q‐type Ca^2+^ inhibitor *ω*‐agatoxin. Experiments in triplicate from three human donors. One‐way RM ANOVA;* P* < 0.05 = *; *P* < 0.01 = **; ns = not significant.

If GLP‐1 inhibits glucagon secretion solely via closure of K_ATP_ channels, membrane depolarization and changes in action potential height (i.e., via the same mechanism by which we propose glucose inhibits glucagon secretion; Zhang et al. (2013)), then GLP‐1 should not influence glucagon secretion when the *α*‐cell membrane potential is “clamped” at depolarized values. We therefore investigated whether GLP‐1 can influence glucagon secretion in a membrane potential‐independent manner by using an experimental paradigm that bypasses any effects of GLP‐1 on *α*‐cell electrical activity. To this end, we performed secretion experiments in the presence of 70 mmol/L extracellular K^+^ ([K^+^]_o_), an experimental condition that depolarizes the *α*‐cell membrane potential to ≈−10 mV (De Marinis et al. 2010). Unexpectedly, GLP‐1 still inhibited glucagon secretion by 50% under these conditions (Fig. 7A). Increasing the glucose concentration also suppressed glucagon secretion by ~50%, suggesting that glucose inhibits glucagon secretion independent of changes in membrane potential (Fig. 7A).

### The glucagonostatic effect of GLP‐1 is mediated by PKA‐dependent inhibition of P/Q‐type Ca^2+^ channels and exocytosis

Glucagon secretion in human *α*‐cells is highly dependent on Ca^2+^ influx through P/Q‐type Ca^2+^ channels, which account for 70% of the whole‐cell Ca^2+^ current (Ramracheya et al. 2010). In 70 mmol/L [K^+^]_o_, application of the P/Q‐type inhibitor *ω*‐agatoxin mimicked the inhibitory effect of GLP‐1 on glucagon secretion (Fig. 7B). Furthermore, GLP‐1 exerted no further inhibitory effect in the presence of *ω*‐agatoxin (Fig. 7B). These data suggest that GLP‐1 inhibits glucagon secretion by modulation of P/Q‐type Ca^2+^ channel activity.

**Figure 8:**
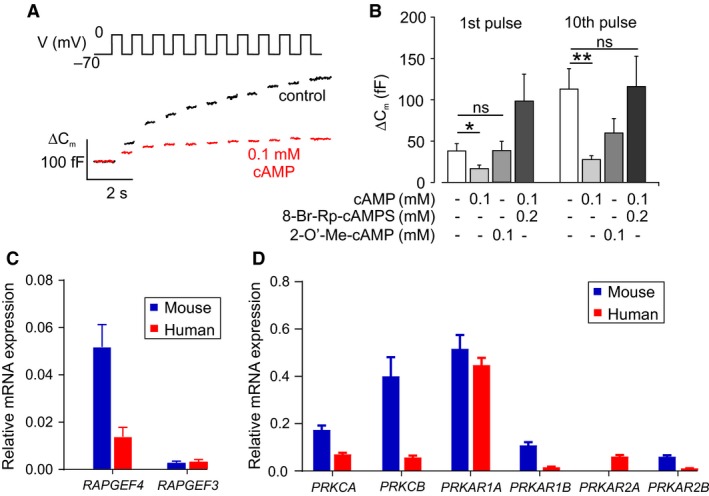
GLP‐1 inhibits exocytosis by a PKA‐dependent mechanism. (A) Monitoring of glucagon exocytosis from dispersed human *α*‐cells by capacitances measurements. Ten membrane potential (*V*_m_) pulses from −70 to 0 mV initiated changes in membrane capacitance (ΔC_m_). Exocytosis was inhibited by inclusion of cAMP in the patch pipette. Glucose concentration is 6 mmol/L. (B) Change in membrane capacitance (ΔC_m_) in response to the 1st and the total increase during the entire train of 10 pulses (Σ_1‐10_) in V_m_. cAMP inhibited the capacitance increase, and this was reversed by application of the PKA inhibitory 8‐Br‐Rp‐cAMPS. The EPAC2 inhibitor 2‐O’‐Me‐cAMP did not reverse the inhibitory effect of cAMP at the 1st pulse (*P* = 0.99) or Σ_1‐10_ pulse (*P* = 0.4564). 25 *α*‐cells from four human donors. Two‐way ANOVA;* P* < 0.05 = *; *P* < 0.01 = **; ns = not significant. Glucose concentration is 6 mmol/L. (C) Expression of *RAPGEF3* and *RAPGEF4* in mouse and human islets. Three mice and four human donors, each measurement in triplicates. (D) Expression of *PRKCA*,*PRKCB*,*PRKAR1A, PRKAR1B, PRKAR2A,* and *PRKAR2B*. Three mice and four human donors, each measurement in triplicates.

Given that P/Q‐type Ca^2+^ channels are important for exocytosis in human *α*‐cells (Ramracheya et al. 2010), and that the effect of GLP‐1 is mediated by cAMP, intracellular application of cAMP should inhibit exocytosis. Exocytosis was monitored by changes in cell capacitance (Gopel et al. 2004), and evoked by a train of 500 ms depolarizations from −70 to 0 mV (Fig. 8A). Under control conditions, this resulted in a biphasic stimulation of exocytosis: the first pulse evoked a large response and subsequent pulses induced progressively smaller capacitance increases. Inclusion of cAMP (0.1 mmol/L) in the intracellular solution reduced the response to the first pulse and the entire train, by ~50% and ~90%, respectively (Fig. 8A).

The inhibitory effect of cAMP on exocytosis was antagonized by the PKA inhibitor Rp‐cAMPS (Fig. 8B), suggesting that cAMP inhibits glucagon secretion via a PKA‐sensitive pathway. EPAC2 is a major second messenger and target for cAMP (de Rooij et al. 1998). We therefore tested the effect of the EPAC2 agonist 2‐O’‐methyl‐cAMP on exocytosis. This EPAC2 agonist did not mimic the effect cAMP: the exocytotic response remained the same as in the absence of cAMP (Fig. 8B). A similar pattern was observed when the analyses were based on the total exocytotic response to the entire train of depolarizations.

The lack of effect of EPAC2 activation in human *α*‐cells contrasts to the strong stimulatory effect observed in mouse *α*‐cells (De Marinis et al. 2010). One explanation for the failure of the EPAC2 agonist to affect exocytosis is that human islet *α*‐cells, unlike their mouse counterparts, express low levels of EPAC2. Indeed, expression of *RAPGEF4* (which encodes EPAC2) is much lower in the human islets used for these experiments than in mouse islets (Fig. 8C), in agreement with RNA‐seq data (Benner et al. 2014). By contrast, the expression of regulatory and catalytic subunits of PKA was the same in mouse and human islets (Fig. 8D).

## Discussion

GLP‐1 agonists and inhibitors of GLP‐1 degradation are major therapies for T2DM (Andersen et al. 2018). GLP‐1 infusions in nondiabetic men have demonstrated that the plasma glucose‐lowering action of GLP‐1 is due to both a reduction in glucagon and increase in insulin secretion (Hare et al. 2010). The regulation of glucagon secretion from the pancreatic *α*‐cells remains obscure even under healthy conditions, with evidence for intrinsic (Zhang et al. 2007), paracrine (Almaca et al. 2016), juxtacrine (Hutchens and Piston 2015), neuronal (Rosario et al. 2016), hormonal (Pipeleers et al. 1985), and hepatic (Kim et al. 2017) modulation. Furthermore, our knowledge of the cellular regulation of glucagon secretion comes from studies of rodent pancreatic islets, with a limited number of studies examining human *α*‐cells (Cabrera et al. 2006; MacDonald et al. 2007; Spigelman et al. 2010). Here, we have investigated the mechanism by which GLP‐1 regulates glucagon secretion from human pancreatic islets. We report that GLP‐1 inhibits glucagon secretion from human islets via a direct action on *α*‐cells, despite the low expression of GLP‐1 receptors. This effect was PKA‐dependent and involved inhibition of P/Q‐type Ca^2+^ channels.

### Paracrine signaling does not mediate GLP‐1 inhibition of glucagon secretion

SST (Hauge‐Evans et al. 2009; Briant et al. 2018b) and insulin (Kawamori et al. 2009) inhibit glucagon secretion. In dispersed rat *α*‐cells, where these influences are removed, GLP‐1 stimulates rather than inhibits exocytosis (Ding et al. 1997). For this reason, GLP‐1 may act by increasing SST or insulin secretion. Consistent with this, we show that human *β*‐ and *δ*‐cells have high expression of the GLP1‐R (Fig. 1), mirroring mRNA data from studies in mouse and human islets (Benner et al. 2014; Adriaenssens et al. 2016; DiGruccio et al. 2016; van der Meulen et al. 2017). However, inhibiting SST or insulin signaling did not abolish the inhibitory effect of GLP‐1 on glucagon secretion in human *α*‐cells, suggesting that GLP‐1 exerts its glucagonostatic effect by a nonparacrine mechanism. We note that S961 appeared to stimulate glucagon secretion in the absence of GLP‐1, which is contrary to previous secretion data in human islets (Elliott et al. 2015). However, we note that we have used a higher concentration of S961 (5 *μ*mol/L), which may have produced a more effective block of the insulin receptor. Indeed, the use of a high concentration seems appropriate given that the interstitial concentration of insulin is very high; it has been estimated that release of a single insulin granule would be sufficient to increase the interstitial insulin concentration to 10 nmol/L (>100‐fold higher than the circulating levels; Rorsman and Ashcroft (2018)). Thus, we conclude that glucagon secretion in human islets may be under partial tonic suppression by basal insulin secretion.

### GLP‐1 inhibition of glucagon may be via direct action on GLP‐1 receptors

Surprisingly, despite the very low expression of GLP‐1 receptors in human *α*‐cells, both at the protein level (Fig. 1) and mRNA level (Tornehave et al. 2008; Benner et al. 2014), it appears that GLP‐1 acts by a direct effect on the *α*‐cell, because the GLP‐1R antagonist exendin (9–39) abolishes the effect of GLP‐1 on glucagon secretion, suggesting that activation of GLP‐1Rs accounts for at least part of the mechanism by which GLP‐1 acts. We note that a recent study reports the development of an *α*‐cell‐specific GLP‐1R KO mouse (*α*GLP‐1R KO mice; Zhang et al. (2018)). They demonstrate that nonfasting glucagon secretion is higher in *α*GLP‐1R KO mice, despite similar insulin and GLP‐1 levels. These data suggest that GLP‐1 is no longer able to inhibit glucagon secretion, supporting our hypothesis. It also supports the presence of GLP‐1R in *α*‐cells, despite an inability to detect them at the protein level or transcriptionally. However, we acknowledge that it should also be considered whether some of the actions of GLP‐1 on glucagon secretion may involve receptors and/or mechanisms other than the canonical GLP‐1 receptor, for example GPR119 (however, recent studies have shown that GPR119 agonism enhanced glucagon secretion; Li et al. (2018)).

### The action of GLP‐1 mimics K_ATP_‐channel closure

Despite its strong and consistent effects on glucagon secretion, GLP‐1 did not affect [Ca^2+^]_i_ in human *α*‐cells. This is similar to what has been observed in mouse islet *α*‐cells (De Marinis et al. 2010).

In line with the weak effects of GLP‐1 on *α*‐cell [Ca^2+^]_i_, there was no suppression in the frequency of firing of action potentials in response to GLP‐1. However, GLP‐1 reduced the amplitude of the action potentials, an effect that was associated with a reduction in the interspike membrane potential. This reduction was small (10 mV), but may be functionally highly significant as exocytosis in human *α*‐cells exhibits a strong dependence on voltage; at voltages below −10 mV, exocytosis decreases by 5% for every mV reduction of the depolarizing command (Ramracheya et al. 2010). In fact, the observed decrease in action potential amplitude predicts a 50% decrease in exocytosis, in close agreement with the suppression of glucagon secretion by GLP‐1 we observed experimentally.

How, then, does GLP‐1 reduce action potential height? The finding that the K_ATP_ channel activator diazoxide was able to counteract the inhibitory effect of GLP‐1 on glucagon secretion (Fig. 6) seemingly suggests that the hormone inhibits glucagon secretion by exerting a glucose‐ or tolbutamide‐like effect (see Zhang et al. (2013)). However, GLP‐1 remained capable of inhibiting glucagon secretion in high [K^+^]_o_. This observation is not consistent with the idea that GLP‐1 mediates its effect via K_ATP_ channel closure, membrane depolarization and reduced action potential height; it instead argues that GLP‐1 must exert (at least part of) its inhibitory effects independent of the K_ATP_ channel. This aspect will be considered below.

### GLP‐1 inhibition of glucagon secretion is mediated by small changes in cAMP

In mouse *α*‐cells, small elevations in cAMP reduce glucagon release by PKA‐dependent inhibition of N‐type Ca^2+^ channels (De Marinis et al. 2010). However, the expression of *Cacna1b* (that encodes the *α*‐subunit of N‐type Ca^2+^ channels) in mouse *α*‐cells is low and it is now believed that this Ca^2+^ current component is mediated by P/Q‐type Ca^2+^ channels (Rorsman et al. 2014).

In this study of human *α*‐cells, we have similarly found that low elevations of cAMP, achieved via application of forskolin, inhibit glucagon secretion. In line with this, we found that cAMP reduced depolarization‐evoked changes in cell capacitance. Furthermore, the PKA inhibitor Rp‐cAMPS could reverse the effect of cAMP on exocytotic capacity and GLP‐1 on glucagon secretion, demonstrating that this effect is PKA‐dependent. We propose that in human *α*‐cells the inhibitory effect of GLP‐1 on glucagon secretion is mediated via activation of PKA, resulting from small elevations in cAMP — an effect that may be facilitated by the low expression of the GLP‐1 receptor.

In mouse islets, high cAMP levels activate glucagon exocytosis via the low affinity cAMP sensor EPAC2 (De Marinis et al. 2010). The lower expression of *RAPGEF4* in human islets may therefore explain why high concentrations of forskolin and application of the EPAC2 agonist 2‐O’‐Me‐cAMP failed to stimulate glucagon secretion and changes in cell capacitance, respectively.

It has been proposed that the stimulation of glucagon secretion at low glucose is — at least in mouse islets — mediated by cAMP/PKA (Elliott et al. 2015; Tengholm and Gylfe 2017). It is therefore of interest that although Rp‐cAMPS abolished the inhibitory effect of GLP‐1, glucagon secretion at 1 mmol/L glucose was unaffected by application of the PKA inhibitor alone (Fig. 4A). This suggests that, at least in human *α*‐cells, secretion of glucagon in 1 mmol/L glucose is not driven by a cAMP/PKA‐dependent mechanism.

### Cyclic AMP‐dependent inhibition of P/Q‐type Ca^2+^ channels explains both effects of GLP‐1 on *α*‐cell electrical activity and glucagon secretion

We suggest that a single mechanism (inhibition of P/Q‐type Ca^2+^ channels) accounts for both the effects on *α*‐cell electrical activity and the suppression of glucagon secretion. These effects are mediated by GLP‐1 binding to the low number of GLP‐1Rs in *α*‐cells, causing a small increase in intracellular cAMP concentration that is just sufficient to activate PKA. This may result in PKA‐dependent phosphorylation of P/Q‐type Ca^2+^‐channel and reduced Ca^2+^ channel activity. The exact mechanism by which PKA inhibits P/Q‐type channels is not clear. The ability of G‐proteins to inhibit Ca^2+^ channels is well‐known (Mintz and Bean 1993; Herlitze et al. 1996). For the low‐voltage activated T‐type Ca^2+^ channel, PKA acts as a “molecular switch”, allowing voltage‐independent inhibition of the channel by G‐protein dimers (Hu et al. 2009). A similar mechanism may exist in human *α*‐cells, whereby PKA permits P/Q‐type Ca^2+^ channel inhibition by G‐proteins that are activated by GLP‐1. We postulate that reduced P/Q‐type Ca^2+^ channel activity explains the suppression of *α*‐cell exocytosis/glucagon secretion.

**Figure 9:**
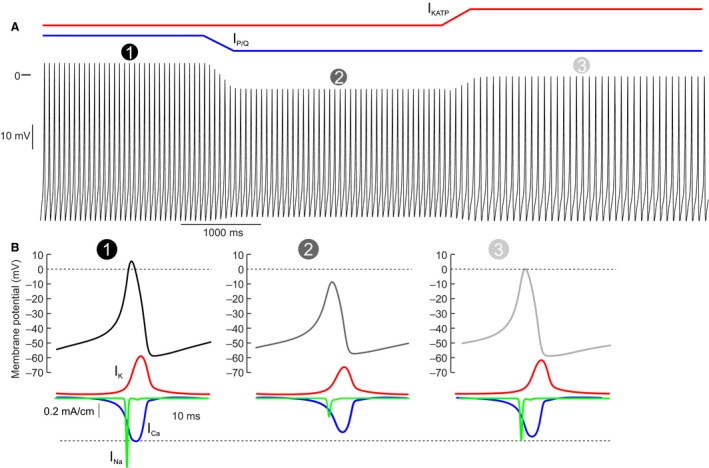
Mathematical model of P/Q‐type Ca^2+^ inhibition in human *α*‐cells. (A) Mathematical model of membrane potential in a human *α*‐cell. The model was simulated under low glucose conditions 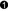. The P/Q‐type Ca^2+^ current was then reduced 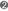, mimicking GLP‐1 application (Fig. 7B). The K_ATP_ current was then increased 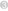, to mimic the action of diazoxide (Fig. 6). (B) The influence of the different simulation conditions on action potential height. P/Q‐type inhibition reduces action potential height 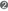, and this is restored by increasing the K_ATP_ current 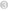. The below traces show the transmembrane K^+^ (red), Na^+^ (green), and Ca^2+^ (blue) currents (I_K_, I_N__a_ and I_C__a_, respectively) underlying the action potential. These simulation data explain why diazoxide can restore glucagon secretion in the presence of GLP‐1.

However, in addition to this effect on exocytosis, inhibition of the P/Q‐type Ca^2+^ channel also causes a decrease in action potential amplitude. In isolated human *α*‐cells, the Ca^2+^ currents constitute 75% of the total voltage‐gated inward current, with the P/Q type Ca^2+^ channels accounting for 70% of the Ca^2+^ current (Ramracheya et al. 2010; Rorsman et al. 2012). A reduced P/Q‐type Ca^2+^ current will result in a lower action potential amplitude, as supported by our mathematical model (Fig. 9A). Importantly, the reduction of action potential height will be associated with reduced activation of the voltage‐gated K^+^ channels involved in action potential. The activation of these channels is voltage‐dependent: the larger the amplitude of the action potential/depolarization, the greater the activation. Thus, the reduction of action potential height due to inhibition of P/Q‐type Ca^2+^ channels will be associated with reduced activation of voltage‐gated K^+^ channels. We emphasize that K^+^ channel activity will drive the membrane potential towards the K^+^ equilibrium potential which is approximately −80 mV. Voltage‐gated K^+^ channels close with a delay upon action potential repolarization. Therefore, a reduction in this current can be expected to result in a depolarization of the membrane potential between two successive action potentials (the interspike membrane potential), a phenomenon also recapitulated by our model.

If GLP‐1 modulates *α*‐cell electrical activity by a K_ATP_ channel‐independent effect, why does the K_ATP_ channel activator diazoxide reverse the inhibitory effect of GLP‐1 on glucagon secretion in human *α*‐cells? We propose and demonstrate with our mathematical model (Fig. 9B) – that a small concentration of diazoxide acts by producing a small repolarization of the *α*‐cell. This provides sufficient reactivation of the voltage‐gated Na^+^ channels, which are almost completely inactivated at the interspike membrane potential in the presence of GLP‐1. The increased Na^+^ current leads to increased spike height, producing greater regenerative activation of voltage‐gated Ca^2+^ channels, and therefore stimulation of glucagon exocytosis. Conversely, GLP‐1, by inhibiting Ca^2+^ channels, reduces spike height and thereby results in reduced regenerative activation of voltage‐gated K^+^ channels and interspike depolarization.

## Conclusions

We have previously proposed that the effects of glucose are mediated by reduced K_ATP_ channel activity (MacDonald et al. 2007; Zhang et al. 2013). This conclusion was based on the finding that low concentrations of diazoxide reverses the inhibitory effect of glucose on glucagon secretion in human islets. However, the in silico analyses presented here indicate that these effects can be interpreted differently, demonstrating the risks associated with overreliance on pharmacological tools. We propose that GLP‐1 exerts its glucagonostatic effect in human islets by an intrinsic (nonparacrine) PKA‐dependent effect, mediated by activation of the few GLP‐1 receptors present in the *α*‐cell plasma membrane, and that culminates in inhibition of the P/Q‐type Ca^2+^ channels. The mechanism is similar to that we have previously documented in mouse islets suggesting it is conserved between different species. The finding that glucose also remains capable of inhibiting glucagon secretion in depolarized islets raises the interesting possibility that hyperglycemia may also inhibit glucagon secretion by a P/Q‐type channel‐dependent process, independent of membrane potential. To extend these data to human islets is also important given the increased clinical use of agents that target the GLP‐1 receptor‐mediated effects and data suggesting that suppression of glucagon secretion contributes significantly to the hypoglycemic effects of these agents.

## Conflict of Interest

The authors declare no conflict of interest.
